# Proto Kranz-like leaf traits and cellular ionic regulation are associated with salinity tolerance in a halophytic wild rice

**DOI:** 10.1007/s44154-021-00016-z

**Published:** 2022-01-27

**Authors:** Miing-Tiem Yong, Celymar Angela Solis, Samuel Amatoury, Gothandapani Sellamuthu, Raja Rajakani, Michelle Mak, Gayatri Venkataraman, Lana Shabala, Meixue Zhou, Oula Ghannoum, Paul Holford, Samsul Huda, Sergey Shabala, Zhong-Hua Chen

**Affiliations:** 1 School of Science, Western Sydney University, Penrith, NSW 2751 Australia; 2 Tasmanian Institute of Agriculture, College of Science and Engineering, University of Tasmania, Hobart, Tasmania 7001 Australia; 3 Plant Molecular Biology Laboratory, M. S. Swaminathan Research Foundation, III Cross Street, Taramani Institutional Area, -600113, Chennai, India; 4 Hawkesbury Institute for the Environment, Western Sydney University, Penrith, NSW 2751 Australia; 5 International Research Centre for Environmental Membrane Biology, Foshan University, Foshan, 528000 China

**Keywords:** Gas exchange, Gene expression, Ion flux, Na^+^ imaging, *Oryza sativa*, *Oryza coarctata*

## Abstract

Species of wild rice (*Oryza* spp.) possess a wide range of stress tolerance traits that can be potentially utilized in breeding climate-resilient cultivated rice cultivars (*Oryza sativa*) thereby aiding global food security. In this study, we conducted a greenhouse trial to evaluate the salinity tolerance of six wild rice species, one cultivated rice cultivar (IR64) and one landrace (Pokkali) using a range of electrophysiological, imaging, and whole-plant physiological techniques. Three wild species (*O. latifolia, O. officinalis* and *O. coarctata)* were found to possess superior salinity stress tolerance. The underlying mechanisms, however, were strikingly different. Na^+^ accumulation in leaves of *O. latifolia*, *O. officinalis* and *O. coarctata* were significantly higher than the tolerant landrace, Pokkali. Na^+^ accumulation in mesophyll cells was only observed in *O. coarctata*, suggesting that *O. officinalis* and *O. latifolia* avoid Na^+^ accumulation in mesophyll by allocating Na^+^ to other parts of the leaf. The finding also suggests that *O. coarctata* might be able to employ Na^+^ as osmolyte without affecting its growth. Further study of Na^+^ allocation in leaves will be helpful to understand the mechanisms of Na^+^ accumulation in these species. In addition, *O. coarctata* showed Proto Kranz-like leaf anatomy (enlarged bundle sheath cells and lower numbers of mesophyll cells), and higher expression of C_4_-related genes (e.g., *NADPME*, *PPDK*) and was a clear outlier with respect to salinity tolerance among the studied wild and cultivated *Oryza* species. The unique phylogenetic relationship of *O. coarctata* with C_4_ grasses suggests the potential of this species for breeding rice with high photosynthetic rate under salinity stress in the future.

**Supplementary Information:**

The online version contains supplementary material available at 10.1007/s44154-021-00016-z.

## Introduction

Salinity tolerance is a polygenetic trait that has evolved multiple times in diverse genera (Bromham et al. 2020) due to various modifications in plant physiological and anatomical traits (Chen and Soltis 2020; Munns et al. 2020a; Solis et al. 2020). Phylogenetic analysis shows that salt-tolerant lineages exhibit a ‘tippy’ pattern (occurring on the tips of the phylogeny rather than internally) (Flowers et al. 2010; Bromham et al. 2020). This may suggest a potential loss or gain of salt-tolerant traits in different species during their evolutionary and ecological adaptation to saline conditions (Bromham et al. 2020; Chen and Soltis 2020; Caperta et al. 2020). Salt tolerance also appears to have evolved more frequently in plants with C_4_ rather than C_3_ photosynthesis (Bromham and Bennett 2014).

The genus *Oryza* (Poales, Poaceae) contains 24 species (Ge et al. 2001), with the Asian rice, *O. sativa,* and African rice, *O. glaberrima,* being staple crops for over half of the global population (Grieve et al. 2019). Cultivated rice is highly susceptible to salinity stress, and a yield penalty occurs at low salinity levels with electrical conductivities (EC) of 3 dS m^− 1^ (Munns et al. 2006; Khatun and Flowers 1995; Lutts et al. 1995). In the cultivated rice, *O. sativa*, salt exclusion from roots, retrieval from the shoots, tissue tolerance (achieved by efficient vacuolar Na^+^ compartmentation and cytosolic K^+^ retention), ROS detoxification, and osmotic adjustment are considered to be the main salinity tolerance mechanisms (Malagoli et al. 2008; Nemati et al. 2011; Kavitha et al. 2012; Kobayashi et al. 2017; Lakra et al. 2018; Oda et al. 2018; Liu et al. 2019). Attempts to increase salinity tolerance have mostly focused on traits found in salt-tolerant genotypes such as Pokkali, Nona Bokra and FL468, that have poor reproductive performance resulting in low yields. Salinity tolerance in these lines is mainly achieved by restricting Na^+^ accumulation in aboveground tissues and by maintaining higher K^+^ contents (Lutts et al. 1996b, a; Prusty et al. 2018; Gerona et al. 2019). However, development of salinity tolerant lines using these landraces has produced plants that also have poor reproductive traits (Solis et al. 2020), suggesting a negative trade-off between salt sensitivity and yield.

To date, only limited numbers of highly salt-tolerant wild *Oryza*  have been reported. One is halophytic *O. coarctata* (KKLL genome)*. *Previous findings have demonstrated that salt-tolerant wild rice species employ tolerance mechanisms different from *O. sativa.* This suggests that salinity tolerance in the genus *Oryza* may have been acquired in multiple, independent, and recent events similar to the trait’s acquisition in other genera (Bromham et al., 2020). In rice, breeding for salinity tolerance using wild relatives has mostly focused on *O. rufipogon* and *O. nivara*, two species that have the same AA genome as cultivated rice (Ganeshan et al. 2016; Wang et al. 2017c). The glycophytic species, *O. officinalis* (EE)*, O. latifolia* (CCDD), and *O. alta* (CCDD) were found to have higher leaf Na^+^ accumulation during salinity treatment (Nakamura et al. 2002; Nishizawa et al. 2015; Prusty et al. 2018; Yichie et al. 2018). Halophytic *O. coarctata* has unique morphological and anatomical features such as thick and waxy leaves that contain salt glands and a differentiated rhizome that adapted to high salinity in coastal areas (Sengupta and Majumder 2010; Rajakani et al. 2021). However, ion homeostasis of photosynthetically active mesophyll tissue of *O. coarctata* in response to high salinity remains unclear.

Leaf Na^+^ exclusion and Na^+^ sequestration are two main mechanisms of plants in response to Na^+^ stress (Hanin et al. 2016). Exclusion is mainly mediated by plasma membrane Salt Overly Sensitive1 (SOS1) antiporters that exclude Na^+^ from mesophyll and retrieval into xylem parenchyma cells via High affinity K^+^ Transporter1 (HKT1) transporters (Hamamoto et al. 2015). The former is controlled by the SOS signalling pathway, which is consists of SOS3, SOS2 and SOS1 mediated by Ca^2+^ and ROS signals (Ji et al. 2013). Na^+^ sequestration mainly carried by Na^+^/H^+^ exchanger1 (NHX1) antiporters from the cytosol to the vacuole (Kronzucker and Britto 2011). Sequestered Na^+^ can be used as a ‘cheap osmoticum’ to increase the salt concentration in vacuoles and  thereby enhance water retention in cell when the external concentration of Na^+^ is high. Moreover, transporters involved in K^+^ retention are also important for maintaining high cytosolic K^+^/Na^+^ ratios (Shabala and Cuin 2008), such as HAK transporters and KEA transporters (Rodríguez-Rosales et al. 2008; Bassil et al. 2011; Shen et al. 2015; Tsujii et al. 2019). The tonoplast and plasma membrane-based proton pumps, V-PPase, V-ATPase and H^+^-ATPase, are also important for providing the H^+^ driving force for Na^+^ transport (Queirós et al. 2009; Zhang et al. 1999). However, the functions of these two salt tolerance mechanisms are not fully explored in wild rice species*.*

*O. coarctata* is found in coastal environments that have daily fluctuations in EC between 20 and 40 dS m^− 1^. *O. coarctata* is the only *Oryza* species with a well-developed, putative, Kranz-like anatomy and has enlarged bundle sheath cells associated with a deep leaf furrow on the adaxial side of the leaf and a pair of longitudinally oriented vascular veins on each leaf ridge (Chatterjee et al. 2016; Rajakani et al. 2019). In C_4_ plants, enlarged bundle sheaths, reduced mesophyll cell/vascular bundle sheath cell size ratios, and a reduced number of mesophyll cells between vascular system in leaves are fundamental structures for C_4_ photosynthesis (Hatch 1987). These features in *O. coarctata* may compensate for the increased distance between the upper mesophyll cells and the main vein due to increased leaf thickness and may contribute to the adaptation to extreme, saline environments. The incorporation of C_4_ photosynthesis into new rice cultivars is suggested as a potential innovative technology towards another Green Revolution to meet future food demand (von Caemmerer et al. 2012). However, the development of C_4_ rice has mostly relied on the introduction of genes from C_4_ grasses (e.g., maize and sorghum) as no C_4_ *Oryza* species has been discovered so far (Wang et al. 2016c). A C_4_ wild *Oryza* species would be ideal to study evolution and function Kranz anatomy, and C_4_ photosynthesis and would facilitate the development of C_4_ cultivated rice.

In this study, we evaluated the salinity tolerance of six wild *Oryza* species, one salt-tolerant landrace and a salt-sensitive cultivar of cultivated rice in the greenhouse. We hypothesized that *O. coarctata* is a C_3_-C_4_ intermediate grass that has salt tolerance linked to the evolution of Kranz leaf anatomy and photosynthesis. Our measurements focused on the response to salinity of typical tolerance indicators (e.g., biomass, Na^+^/K^+^ ratio), photosynthesis, ion transport [as assessed by microelectrode ion flux estimation (MIFE)], confocal imaging of reactive oxygen species (ROS) and Na^+^ concentrations in mesophyll cells, and expression of genes associated with ion transporters and C_4_-related proteins. Our data suggest that three wild species, *O. latifolia, O. officinalis*, and *O. coarctata,* are all highly tolerant of salinity stress; however, this was achieved by employing different mechanisms. All these species accumulated significant amounts of Na^+^ in the shoot. However, Na^+^ accumulation in mesophyll cells was only observed in *O. coarctata*, while *O. officinalis* and *O. latifolia* may allocate Na^+^ to other leaf/shoot tissues. It is concluded that the above traits may be used as a potential target for breeding salt-tolerant rice cultivars.

## Results

Although salinity stress had a significant, negative effect on the performance of all plants, a large variation in stress tolerance was observed amongst the various rice lines. The relative values of the growth and physiological parameters of these lines were ranked using the homogenous groups assigned by DMRTs (Supplementary Table S2). According to the ranking, overall performance under saline conditions was (from poorest to greatest) as follows: *O. longiglumis* < IR64 < *O. australiensis* < *O. rufipogon* < *O. latifolia* = *O. officinalis* < Pokkali < *O. coarctata.*

**Fig. 1:**
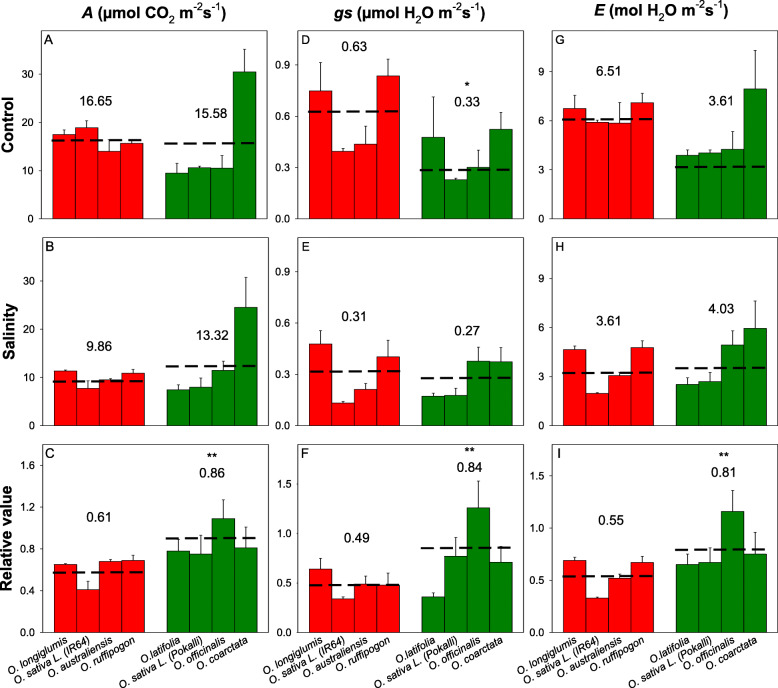
Effects of 100 mM NaCl treatment on leaf gas exchange parameters of wild and the cultivated rice after six weeks of salinity stress**.** (**A-C**) net CO_2_ assimilation rate, *A*; (**D-F**) stomata conductance, *g*_*s*_; and (**G-I**) transpiration rate, *E.* For each species, left bar (red) represents the sensitive species group and right bar (green) represents the tolerant species group. * and ** indicates significant difference found between sensitive and tolerant groups at *P* < 0.05 and *P* < 0.01 respectively, using independent sample t-test. The number and dashed line indicate the mean value of each group. The data are means and the error bars indicate the standard errors

Significant reductions in biomass due to salinity stress were found in *O. longiglumis*, IR64, *O. australiensis,* and *O. rufipogon* (*P* < 0.05; salinity-sensitive lines) (Supplementary Figs. 1) but not for *O. latifolia,* Pokkali, and *O. officinalis* and *O. coarctata* (salt-tolerant lines); *O*. *coarctata* even showed a small (although non-significant) increase in biomass under salinity treatment*.* Tiller numbers and plant heights were not significantly affected by salinity treatment in any of the species (Supplementary Figs. S1). In terms of photosynthetic properties, total chlorophyll, net CO_2_ assimilation rates (*A*), stomata conductance (*g*_*s*_), and transpiration rates (*E*) of salinity stressed plants were significantly (ANOVA, treatment effect, *p* < 0.001) reduced compared to those in the control plants. Significant reductions in the total chlorophyll contents, CO_2_ assimilation rates (*A*), stomata conductance (*g*_*s*_), and transpiration rates (*E*) were found in sensitive species- *O. longiglumis* (except *g*_*s*_ and *E*, *P* > 0.05)*,* IR64, and *O. rufipogon* (Figs. 1). Compared to the other rice species, *O*. *coarctata* showed unique photosynthetic parameters such as significantly higher *A* and water use efficiency (*WUE*) and lower intercellular CO_2_ concentrations (*Ci*); these parameters were not affected by the salinity treatment.

### Ion transport in leaf mesophyll differ dramatically between *O. coarctata* and other *Oryza* species

**Fig. 2:**
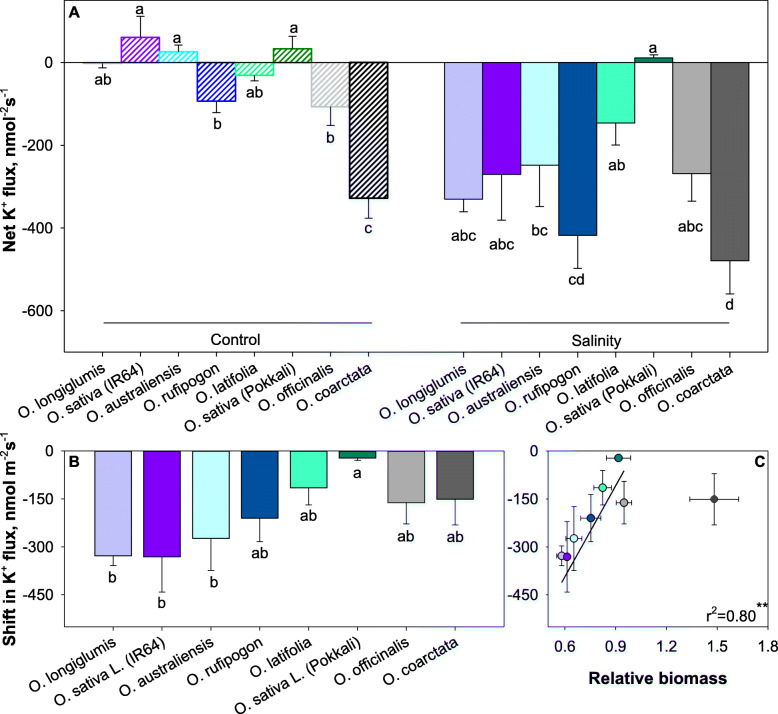
Linking salinity tolerance of wild and cultivated rice to net K^+^ ion fluxes of mesophyll cells (**A**) after 42 days of 100 mM NaCl treatment. The shift in K^+^ flux was the flux difference between flux from salinity-stressed and control samples (**B**). Pearson analysis of shift in K^+^ flux with relative biomass (**C**). Data are mean values and the error bars indicate the standard errors. Different lowercase letters indicate significant differences at P < 0.05 for each Duncan group

Steady-state K^+^ fluxes from leaf mesophyll were positive (net K^+^ uptake) in salt-sensitive *O. australiensis* and *O. longiglumis* and both cultivated *O. sativa* genotypes (Fig. 2A). The other species showed a small, net K^+^ effluxes of 0 to − 100 nmol m^− 2^ s^− 1^ except for *O. coarctata* that had much higher net, initial, K^+^ efflux (−328 nmol m^− 2^ s^− 1^). When plants were grown under saline conditions, steady-state mesophyll K^+^ fluxes were drastically shifted towards negative values (net K^+^ loss; Fig. 2B) by 200 to 300 nmol m^− 2^ s^− 1^ (Fig. 2C). *O. coarctata* was an exception and showed no statistically significant difference in mesophyll K^+^ fluxes between control- and salt-grown plants. When *O. coarctata* was omitted from the analysis, a strong positive (r^2^ = 0.8) correlation between salinity-induced shift in mesophyll K^+^ fluxes and relative plant growth under saline conditions was found (Fig. 2C).

**Fig. 3:**
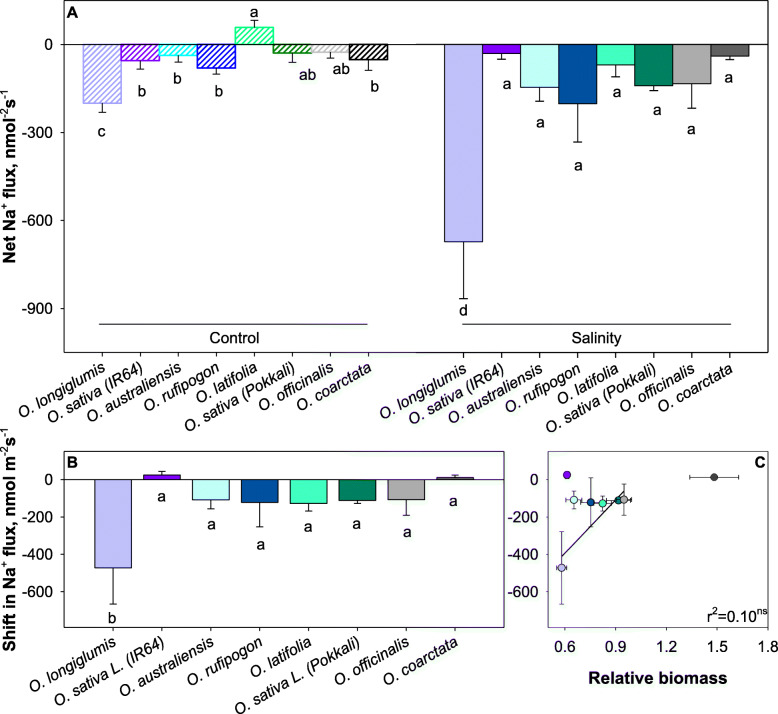
Linking salinity tolerance of wild and cultivated rice to net Na^+^ ion fluxes of mesophyll cells. Salinity treatment was applied at 100 mM NaCl for 42 days. (**A**). Shift in K^+^ flux was the flux difference between flux from salinity stressed and control samples (**B**). Pearson analysis of shift in Na^+^ flux with relative biomass (**C**). Data are mean values and the error bars indicate the standard errors. Different lowercase letters indicate significant differences at *P* < 0.05 for each Duncan group

Net Na^+^ fluxes were around zero in control-grown plants and slightly negative under saline conditions (Fig. 3A). Salinity-induced shifted in net Na^+^ fluxes were about −150 nmol m^− 2^ s^− 1^ (Fig. 3B) and did not correlate with relative plant performance (r^2^ = 0.10; not significant; Fig. 3C).

**Fig. 4:**
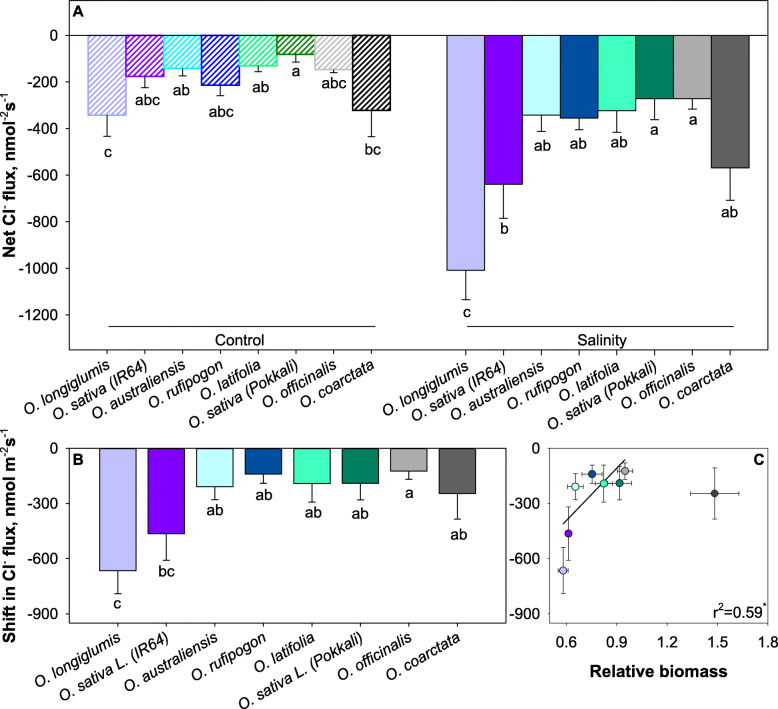
Linking salinity tolerance of wild and cultivated rice to net Cl^−^ ion fluxes of mesophyll cells. Salinity treatment was applied at 100 mM NaCl for 42 days. (**A**) Shift in Cl^−^ flux was the flux difference between flux from salinity stressed and control samples. (**B**) Pearson analysis of shift in Na^+^ flux with relative biomass. (**C**) Data are means and the error bars indicate the standard errors. Different lowercase letters indicate significant differences at *P* < 0.05 for each Duncan group

Steady-state mesophyll Cl^−^ fluxes ranged between −50 and −200 nmol m^− 2^ s^− 1^ (net efflux) in control and increased by 2 to 3-fold under saline conditions (Fig. 4A). Salinity-induced changes in Cl^−^ fluxes were strongest in the salt-sensitive cultivars (Fig. 4B) and correlated positively with plant performance (r^2^ = 0.59; Fig. 4C). Again, *O. coarctata* was an exception from this finding.

### Salt-tolerant *Oryza* species had higher ROS production and lower Na^+^ accumulation in leaf and mesophyll cells

**Fig. 5:**
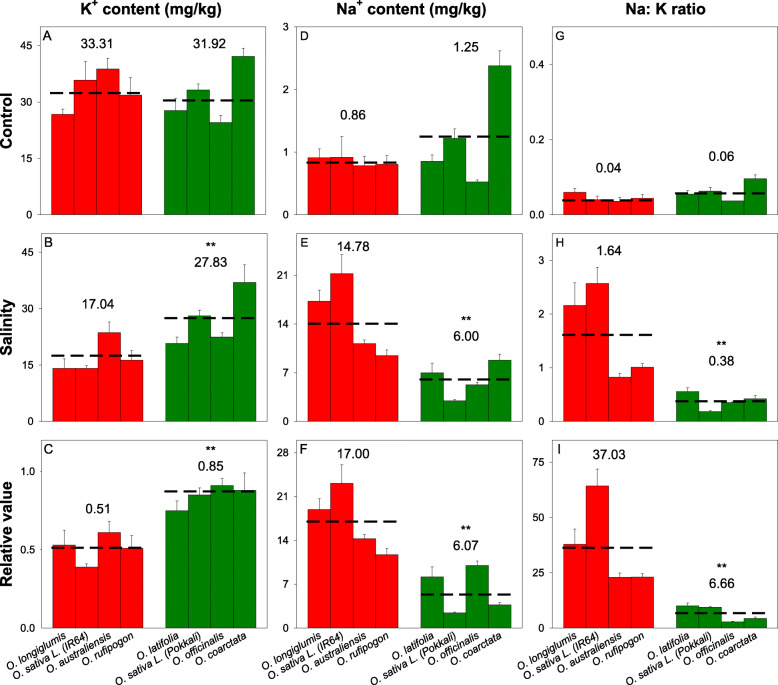
Leaf ion contents in six wild and two cultivated rice lines after 6 weeks of 100 mM salinity treatment. (**A-C**) leaf K^+^ contents, (**D-F**) leaf Na^+^ contents and (**G-I**) leaf Na^+^/K^+^ ratios. Data are mean values and the error bars indicate the standard errors. * and ** indicates significant difference found between sensitive and tolerant groups at *P* < 0.05 and *P* < 0.01, respectively, using independent sample t-test. The number and dashed lines indicate the mean values of each group. The data are means and the error bars indicate the standard errors

In response to salinity stress, all species had a significant reductions in leaf K^+^ contents and increases in leaf Na^+^ contents (Figs. 5, ANOVA, treatment effect, *p* < 0.001). The sensitive species had the highest reduction in shoot K^+^ content and increase in shoot Na^+^ (ANOVA, treatment*species effect, p < 0.001). Few salt-tolerant wild rice (mostly species with C genome) have been reported with higher Na^+^ accumulation in shoots compared to the salt-tolerant *O. sativa* lines. This was also observed with our leaf Na^+^ contents. Na^+^ contents (Figs. 5D–F) and Na^+^/K^+^ ratios (Figs. 5G–I) in the salt-tolerant wild species (*O. latifolia*, *O. officinalis,* and *O. coarctata*) were all significantly higher than the salt-tolerant cultivated genotype Pokkali. Leaf Na^+^/K^+^ ratios in the salinity-sensitive *O. sativa* lines were significantly increased after prolonged salinity stress. Although there were small increases in this ratio for the tolerant lines, the increase was only significant (t-test, *P* < 0.05) for *O. latifolia* (Figs. 5G-H). The leaf Na^+^ (negative; r^2^ = 0.74, P < 0.05), K^+^ (positive; r^2^ = 0.80; *P* < 0.01) and Na^+^/K^+^ ratios (negative; r^2^ = 0.74, P < 0.05) were strongly correlated with relative biomass (Supplementary Table S3).

**Fig. 6:**
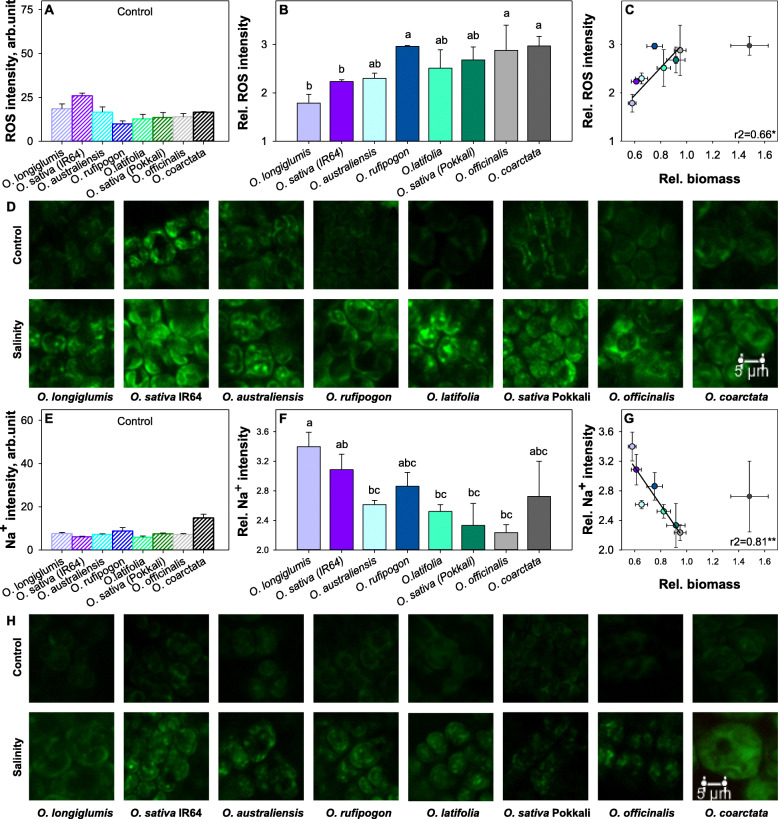
Linking salinity tolerance of wild and cultivated rice to accumulation of reactive oxygen species (ROS) and Na^+^ in leaf mesophyll cells after 42 days of 100 mM NaCl treatment: (**A & E**) ROS & Na^+^ fluorescence in the mesophyll of control plants; (**B & F**) relative ROS and Na^+^ fluorescence in the mesophyll of salinity-stressed and control plants; (**C & G**) Pearson correlation of relative ROS production and Na^+^ fluorescence and relative biomass; (**D & H**) representative image of ROS and Na^+^ in mesophyll. The scale bars = 5 μm. Data are means and the error bars indicate the standard errors. Different lowercase letters indicate significant differences at *P* < 0.05 for each Duncan group

After six weeks of salinity stress, leaf mesophyll cells accumulated significant amounts of ROS (Figs. 6A–D). The ROS contents in leaf mesophyll cells of most species/cultivars ranged between 13 and 19 (arbitrary) units in control-grown plants with the highest ROS accumulation (26 units) found in IR64 and the lowest (10 units) in *O. rufipogon* (Fig. 6A). Surprisingly, the salinity tolerant species showed higher ROS accumulation in mesophyll after the salinity stress (Fig. 6B). The analysis indicated that ROS accumulation was significantly correlated to the following salinity tolerance indicators: biomass (positive, r^2^ = 0.66, P < 0.05), Na^+^/K^+^ ratio (negative, r^2^ = 0.75, P < 0.05), mesophyll Na^+^ fluorescence (negative, r^2^ = 0.56, P < 0.05) and net Cl^−^ flux (positive, r^2^ = 0.79, P < 0.01). *O. coarctata* produced higher ROS concentrations in mesophyll cells and maintained the largest relative biomass in response to salinity treatment (Fig. 6B).

To further confirm whether the Na^+^ was stored in the mesophyll cells of salt-tolerant wild rice species, Na^+^ accumulation in mesophyll cells was evaluated using confocal imaging. The imaging analysis showed a similar trend to that found in leaf Na^+^ contents with mesophyll Na^+^ fluorescence decreasing along with the increase of salt tolerance in those rice species (Figs. 6E–H). The mesophyll Na^+^ fluorescence was moderately correlated to the leaf Na^+^ content (positive, r^2^ = 0.65, P < 0.05) (Supplementary Table S3) and highly correlated to biomass (negative, r^2^ = 0.81, P < 0.01) (Fig. 6G). However, among the tolerant group, only *O. coarctata* had significantly higher mesophyll Na^+^ accumulation. No difference was found between *O. latifolia, O. officinalis,* and Pokkali.

### Salinity stress-induced expression of ion transporter genes reveals stronger ion homeostasis ability in salinity tolerant *Oryza* species

**Fig. 7:**
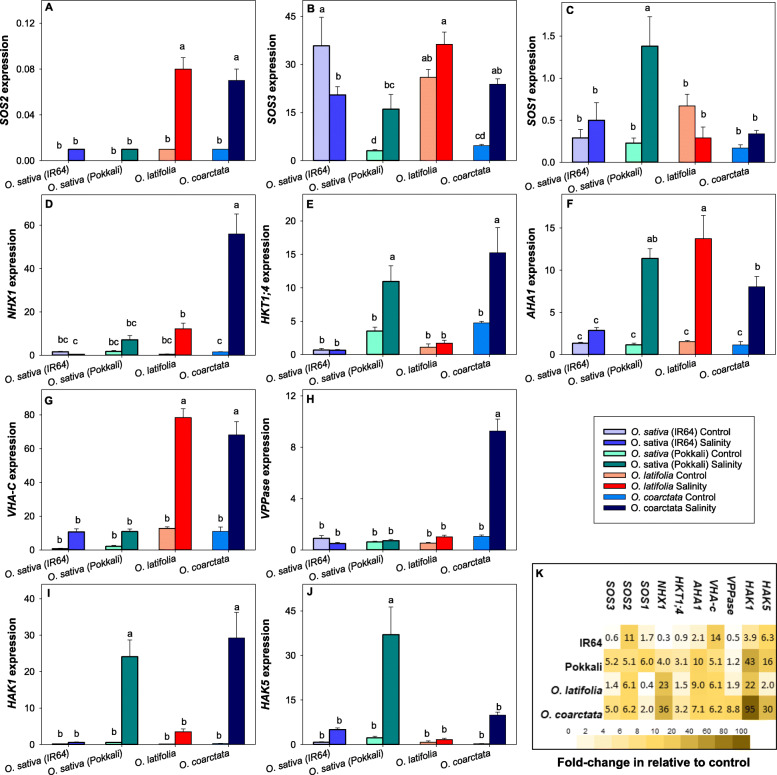
Expression of salinity tolerance-related genes in the leaf of wild and cultivated rice after 42 days of 100 mM NaCl treatment. (**A**) *SOS2,* (**B**) *SOS3,* (**C**) *SOS1,* (**D**) *NHX1,* (**E**) *HKT1;4,* (**F**) *AHA1,* (**G**) *VHA-C,* (**H**) *VPPase*, (**I**) *HAK1* and (**J**) *HAK5.* (**K**) Fold change of genes after salinity treatment is relative to the control. Data are mean of 3 technical replicates (each containing at least 3 technical replicates) and the error bars indicate the standard errors. Different lowercase letters indicate significant differences at *P* < 0.05 for each Duncan group

We then evaluated the expression of ten salinity tolerance-related genes of two tolerant wild species (*O. latifolia, O. coarctata*), and salt-tolerant (Pokkali) and salt-sensitive (IR64) lines of cultivated rice*. sativa*. The selected genes included Na^+^ transporters (*NHX1*, *SOS1*, *HKT1;4*), K^+^ transporters (*high affinity potassium transporters* [*HAK1*], *HAK5*), proton pumps (*plasma membrane H*^*+*^*-ATPase* [*AHA1*], *V-type H*^*+*^*ATPase subunit C* [*VHA-C*]*,* and *vacuolar PPase* [*VPPase*]), and Ca^2+^ signaling proteins (*SOS2, SOS3*). Two-way ANOVA sindicated a significant difference of expression of each gene between species in response to the prolonged salinity treatment (Two-way ANOVA, main effects and species×treatment effects all < 0.001). Expression of the SOS signaling genes- *SOS2*and *SOS3,* were upregulated in all species except *SOS3* expression in salt-sensitive IR64 (Figs. 7A-B). Relative to the control, Na^+^ and K^+^ transporter and proton pump genes were mostly upregulated in the salinity-tolerant wild rice species, *O. coarctata*, *O. latifolia,* and the cultivated rice, Pokkali (Figs. 7C-E). *OlSOS1* was the only transporter gene downregulated in the salt-tolerant species. The salt-sensitive IR64 had the highest numbers of genes (*OsNHX1*, *OsHKT1;4 & OsVPPase*) downregulated in response to prolonged salinity treatment (Fig. 7K).

Expression of the vacuolar Na^+^ compartmentation-related transporter, *NHX1,* was significantly higher in both wild salt-tolerant species (Fig. 7K). On the other hand, the Na^+^ exclusion-related transporter, *SOS1,* was significantly higher in salt-tolerant *O. sativa*. Correspondingly, the expression of the tonoplast proto pumps, *VHA-C* and *VPPase* (only *O*. *coarctata* significantly upregulated), was also higher in the wild tolerant species. This finding supported the importance of Na^+^ compartmentation in both wild species, which had shown higher leaf Na^+^ content and mesophyll Na^+^ compared to the salt-tolerant cultivated rice Pokkali*.* Na^+^ accumulation in *O. coarctata’s* mesophyll was further evaluated in response to direct 50 mM and 100 mM NaCl treatments on exposed mesophyll tissue with the epidermal layer removed for 2 h and in response to 1 week of 100 mM NaCl hydroponic solution (Supplementary Fig. 2). Consistently, the results showed significant Na^+^ accumulation in the vacuole based on the area and shape of the Na^+^ fluorescence in the cell. The Na^+^ fluorescence in the mesophyll cell chloroplasts in response to 1 week of 100 mM NaCl hydroponic solution was surprisingly low compared to the control. The increase in vacuole Na^+^ fluorescence of mesophyll cells with 100 mM NaCl treatment was 5 times higher than mesophyll with 50 mM NaCl treatment. The result suggested that Na^+^ compartmentation is the main strategy of salinity defense in mesophyll cells.

K^+^ inward transporters, *HAK1 & HAK5,* were overall upregulated in all species. The expressions were significantly higher in *O. coarctata* and Pokkali. The fold-change of *HAK1* expression was higher than the fold-change of *HAK5* in all tolerant species. The fold-change *HAK1* was correlated to the leaf K^+^ content (Figs. 5A-C) and biomass (Supplementary Fig. 1), which is highest in *O.coarctata*, followed by Pokkali, *O. latifolia* then IR64.

### *O. coarctata* has a proto Kranz-like anatomy, higher photosynthetic rate, and highly expressed C_4_ photosynthesis-related genes

**Fig. 8:**
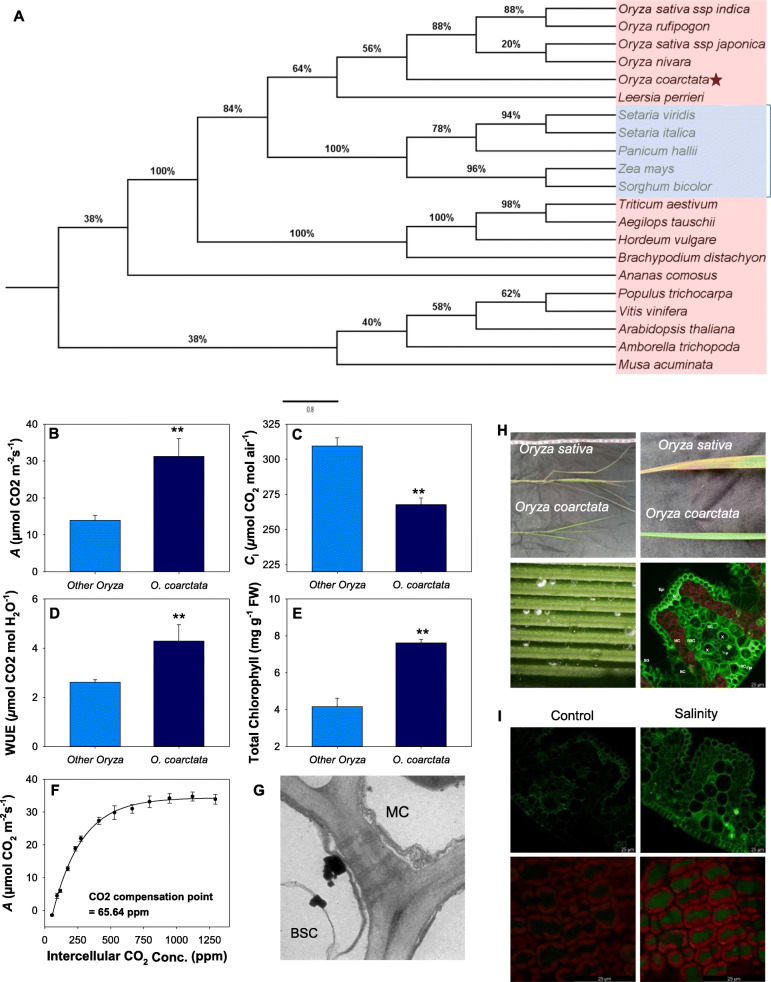
Phylogenetic, morphological and photosynthetic evidence of *O. coarctata* being a C_3_-C_4_ intermediate. (**A**) Maximum likelihood tree showing the phylogenetic relationship of PEPC, a C_4_ key enzyme, in C_3_ (red) and C_4_ (blue) species. The percentages of trees from 1000 bootstrap replications in which the associated taxa clustered together are shown next to the branches. (**B-E**) Leaf gas exchange characteristics of *O. coarctata* compared to other *Oryza* species used in this study. (**F**) Photosynthesis dependence on intracellular CO_2_ curve fitting a C_4_ type photosynthesis model species. (**G**) Leaf ultrastructure showing the presence of plasmodesmata connecting mesophylle and bundle sheath cells. (**H**) Shoot and leaf morphology of *O. sativa* and *O. coarctata*, salt secretion on leaf surface of *O. coarctata*, and Na^+^ localization from leaf cross section of *O. coarctata*. Epi- epidermis, MC- mesophyll cells, BSC- bundle sheath cells, X- xylem, P- phloem, BC-bulliform cells, ST- sclerenchymatous thickening and SG- salt glands. (**I**) Na^+^ accumulation and chlorophyll fluorescence in a leaf in response to high salinity stress in *O. coarctata*.. Curve was fitted with a 3-parameters exponential rise to maximum [Net CO_2_ assimilation = −1.191 + 4.628 × (1-exp(− 4.533 × CO_2_ concentration) with a calculated CO_2_

*O. coarctata* showed significant photosynthetic capacity in the control and salinity stress in the greenhouse trial (Fig. 1). To evaluate whether *O. coarctata* is a C_4_ or a C_3_-C_4_ intermediate species, we first constructed a phylogenic tree of a C_4_-related gene, *phosphoenolpyruvate carboxylase* (*PEPC*) , for a group of C_3_ and C_4_ plants. The phylogeny showed that this gene in *O. coarctata* is phylogenetically closer to those in C_4_ grasses such as *Setaria*, maize, and *Sorghum* compared to other C_3_ species in the *Oryza* genus (Fig. 8A).

Overall, *O. coarctata* had significantly (*P* < 0.01) higher total chlorophyll contents, CO_2_ assimilation rates (*A*), WUEs, and significantly lower intracellular CO_2_ concentrations (*C*_*i*_) compared to the six other *Oryza* species (Figs. 8B–E). The *A/Ci* curves indicate that *O. coarctata* was highly responsive to increasing CO_2_ concentration with CO_2_ compensation points at 64.5 μmol CO_2_ m^− 2^ s^− 1^ (3-parameter exponential curve) (Fig. 8F) and 50.4 μmol CO_2_ m^− 2^ s^− 1^ with a linear fitting of the first 6 points. The photosynthetic characteristics showed the *O. coarctata* may not be a C_4_ rice.

Leaf anatomy was investigated to confirm if *O. coarctata* possesses proto-Kranz or Kranz anatomy. Under transmission electron microscopy (TEM), plasmodesmata connections between mesophyll and vascular bundle sheath cells were observed (Fig. 8G). The cross-sections of *O. coarctata* leaves showed larger vascular bundle sheath cells relative to mesophyll cells, higher numbers of chloroplasts in the vascular bundles, and lower numbers of mesophyll cells between veins. The leaves have distinct adaxial surfaces that have a high density of ridges and furrows (Fig. 8H). Each ridge contains a vascular bundle system, and salt glands for salt exclusion are found on the surface of the furrows. The number of mesophyll cells between each adjacent vascular vein is significantly lower in *O. coarctata* than reported in *O. sativa* (Chatterjee et al. 2016). Confocal imaging analysis showed a strong Na^+^ signal in the epidermal layer, salt glands, leaf veins, and mesophyll cells (Fig. 8I).

**Fig. 9:**
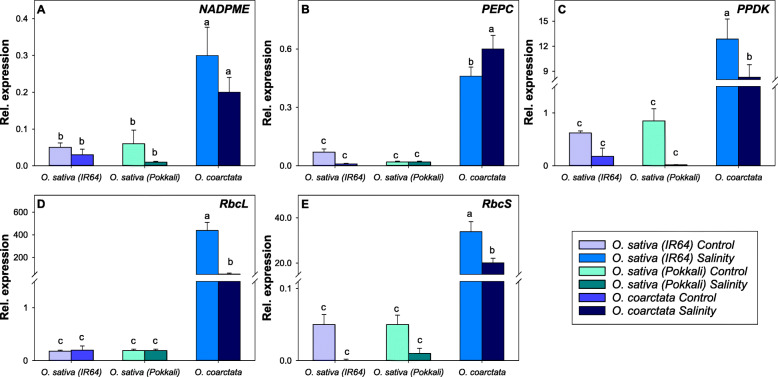
Molecular evidence for *O. coarctata* being a C_3_-C_4_ intermediate. Effect of salinity on the following C_4_ photosynthesis-related genes in *O. coarctata,* Pokkali and IR64: (**A**), *NADP-dependent malic enzyme* (*NADPME*), (**B**) *PEPC,* (**C**) *PPDK,* (**D**) *RBCL*, and (**E**) *RBCS.* Data are means and the error bars indicate the standard errors. Different lowercase letters indicate significant differences at *P* < 0.05 for each Duncan group

Further, we compared the expression of five C_4_ photosynthesis-related genes in *O. coarctata*, IR64, and Pokkali in the control and salinity treatments (Fig. 9). The results indicate that expression of these genes was significantly higher (up to 800-fold for *ribulose-bisphosphate carboxylase* [*RBCL*]) in both control and salinity stressed *O. coarctata* as compared to those in *O. sativa*. *PEPC* was the only gene upregulated in *O. coarctata* after the salinity stress while *pyruvate phosphate dikinase* (*PPDK*)*, RBCL,* and *ribulose-1,5-bisphosphate carboxylase/oxygenase small subunit* (*RBCS*) were significantly downregulated after salinity stress.

## Discussion

More than 10,000 years ago, ancient humans began to consume grains of *O. rufipogon* that grew in the marshes throughout Asia (Sweeney and McCouch 2007). Continuous domestication for desirable traits has slowly caused *O. rufipogon* to evolve into *O. sativa*, a staple crop for billions of people worldwide (Kovach et al. 2007; Sweeney and McCouch 2007). However, after almost 8000 years of cultivation, *O. sativa* is estimated to only have conserved 20% of the genetic diversity of wild *Oryza* (Zong et al. 2007; Palmgren et al. 2015). This narrow genetic diversity is the major constraint on breeding salinity-tolerant rice lines (Waziri et al. 2016; Chen et al. 2020). Thus, investigating gene expression, ion homeostasis, Na^+^ transport between the vascular system and mesophyll tissue in salt-tolerant, wild rice species may enable the identification of new mechanisms that contribute to salt tolerance.

### Salt-tolerant wild rice exhibits distinct leaf tissue tolerance to Na^+^ and K^+^ transport compared to *O. sativa*

Salt-tolerant *O. sativa* generally employs Na^+^ exclusion to avoid high Na^+^ accumulation in aboveground tissues. This trait likely results from artificial selection for Na^+^ exclusion and may account for the poor tissue tolerance to Na^+^ in this rice species (Lutts et al. 1996b, a; Prusty et al. 2018; Gerona et al. 2019). In this study, we evaluated the performance of six wild *Oryza* species with two lines of cultivated rice after prolonged stress at a moderate salinity level of 10 dS m^− 1^. We found *O. coarctata*, *O. officinalis,* and *O. latifolia* were highly tolerant of salinity stress. However, only the halophytic *O. coarctata* showed high Na^+^ accumulation in mesophyll cells when grown under salt stress among salt-tolerant species (Figs. 6), suggesting that the other salt-tolerant wild rice species, *O. officinalis,* and *O. latifolia*, avoid Na^+^ accumulation in mesophyll cells.

*SOS2* and *SOS3* were first identified as key components of the salt overly sensitive pathway to exclude Na^+^ by activating SOS1 (Ji et al. 2013). Other than SOS1, the Ca^2+^-activated SOS2/SOS3 complex was also reported to activate/upregulate tonoplast Ca^2+^ exchangers (CAXs), the internal membrane Na^+^ antiporters, NHX1, NHX2 and NHX4, and the plasma membrane and tonoplast proton pumps,- AHA, V-ATPase, and VPPase (Huertas et al. 2012; Che-Othman et al. 2017; Yang et al. 2019). Expression of these transporter genes (*SOS1, NHX1, AHA, VHA-C,* and *VPPase*) was upregulated in the halophytic O. *coarctata* and mostly upregulated in salt-tolerant Pokkali and *O. latifolia*. SOS1, HKT1;4, and NHX1 are important Na^+^ transporters responsible for Na^+^ exclusion, compartmentation, and retrieval (Wu et al. 2018). The expression of *SOS1* in *O. coarctata* and *O. latifolia* was consistently low and was not affected by six weeks of salt treatment (Figs. 7C, K). Leaf Na^+^ contents in both species were relatively higher than Pokkali (Figs. 5D-F), suggesting that Na^+^ exclusion may not be a main defense mechanism for salt tolerance in mesophyll cells of these wild rice species. In addition, *O. coarctata* and *O. latifolia* had significantly higher *NHX1* expression after salinity stress compared to the control and the two cultivated rice lines (Figs. 7D, K). *O*. *coarctata*’s mesophyll showed a preference to compartmentalise Na^+^ in the vacuole and regulate apoplastic Na^+^ after the salinity treatment (Supplementary Figs. 2), indicating a key role for NHX1 and HKT1;4 in the leaf salt tolerance of *O. coarctata*. *O. latifolia* is a member of *O. officinalis* complex, which is well known for its resistance to various biotic and abiotic stresses (Shenton et al. 2020). In this study, mesophyll Na^+^ in *O. latifolia* was surprisingly low. Given the high leaf Na^+^ content found in *O. latifolia,* higher relative mesophyll Na^+^ fluorescence was expected compared to Pokkali. Na^+^ homeostasis strategies in leaves of salt-tolerant *O. latifolia* require further investigation.

We previously reported a significant, positive correlation between net K^+^ flux and salinity tolerance in *O. sativa* (Yong et al. 2020). Here, except for *O. coarctata,* we extended this finding to wild *Oryza* species (Figs. 2). Our results show a significant, strong, and positive correlation between net K^+^ flux and biomass (Fig. 2E). In addition, net K^+^ flux was also significantly and positively correlated with mesophyll Na^+^, leaf Na^+^ contents, and leaf Na^+^/K^+^ ratios (Supplementary Table S3). K^+^ is the macronutrient responsible for the coordination of more than 50 enzymes in plants (Shabala and Cuin 2008). Stress-induced K^+^ leakage from the tissue was reported in plants due to salinity (Liu et al. 2017a), hypoxia (Wang et al. 2017a), drought (Mak et al. 2014), and high light (Babla et al. 2020). This enables the operation of the GORK channels as a master switch of the cell metabolism, thus adjusting intracellular K^+^ homeostasis to altered environmental conditions (Adem et al. 2020). Also, the K^+^ efflux may be conducted by non-selective cation channels (Zepeda-Jazo et al. 2008). These ion channels may be key factors involved in K^+^ leakage in the sensitive wild rice species in our study. Alternatively, K^+^ can be actively transported into cells through HAK transporters, such as some key members (OsHAK1, OsHAK5, and OsHAK21) of this gene family in rice (Mangano et al. 2008; Chen et al. 2015; Shen et al. 2015; Nieves-Cordones et al. 2017; Feng et al. 2020). *HAK1* and *HAK5* were upregulated in both *O. sativa* and wild species. The *HAK1* expression matched the results of relative biomass and K^+^ content of four species. *HAK5* expression was also highest in the most tolerant species *O. coarctata* and Pokkali but lowest in *O. latifolia*.

### *O. coarctata *is an outlier of salt tolerance among *Oryza* species

The gas exchange required for photosynthesis cannot be maintained without balancing ions, organic compounds, and water contents being maintained within species-specific tissue tolerances under salinity stress (Shabala and Cuin 2008; Mishra et al. 2020; Munns et al. 2020b). In cultivated rice, photosynthetic activities are negatively correlated with the magnitude and duration of salinity stress (Yeo et al. 1985; Radanielson et al. 2018). In this study, *O. coarctata* showed a significantly greater relative biomass after salinity stress as compared to the other species (Figs. S1). In comparison to the other *Oryza* species, *O. coarctata* exhibited the highest *A*, WUE, and total chlorophyll contents in both the control and the salt-treated plants; none of these parameters was significantly affected by salinity stress (Figs. 1 and Supplementary Figs. S1), indicating remarkable adaption of photosynthetic traits to the saline condition in this species. In addition, across the six key physiological parameters that were significantly correlated with relative biomass (Figs. 2 and 4), *O. coarctata* was a consistent outlier compared to the studied glycophytic species, and its salinity tolerance did not show links with net K^+^ and Cl^−^ efflux nor to ROS production and Na^+^ fluorescence. Thus, salinity tolerance of *O. coarctata* must be determined by other mechanisms.

Confocal imaging of Na^+^ of *O. coarctata* revealed Na^+^ fluorescence in the salt glands, the vascular tissues, and in epidermal tissues is higher than that in mesophyll tissues (Fig. 6F). Therefore, most of the Na^+^ may be transferred through the plant to the salt glands without passing through mesophyll cells, the major cell type in which photosynthesis occurs. This was validated by direct treatment of the mesophyll tissue of *O. coarctata* with the epidermis removed with 50 mM and 100 mM NaCl. The directly stressed mesophyll showed obvious accumulation of Na^+^ around or within the chloroplasts (Supplementary Figs. S2). This was not found in leaf samples with intact epidermis from plants subjected to one month of salinity treatment in hydroponic solution. Thus, *O. coarctata* increases Na^+^ accumulation around the mesophyll tissue thereby maintaining photosynthesis (Figs. 1); this has also been found in other plant species such as chickpea (Kotula et al. 2019; Kotula et al. 2020).

### Linking high photosynthetic capacity to high salt tolerance in *O. coarctata*

There are increasing numbers of reports on the potential for the genetic engineering of C_4_ photosynthesis into C_3_ crops such as rice to improve their productivity and stress tolerance (Wang et al. 2017b; Ermakova et al. 2020). Evolutionarily, the transition from C_3_ to C_4_ photosynthesis was closely related to ambient CO_2_ concentration and temperature (Sage 2004; Edwards et al. 2010), which may have been a stepwise conversion of cellular biochemistry and a few key anatomical structures (Wang et al., 2017a). Three C_3_-C_4_ intermediate types, proto-Kranz, C_2_-C_2_+ photosynthesis, and C_4_-like photosynthesis, have been proposed based on the mesophyll cell numbers between veins, the size of bundle sheath cells, the amount and coordination of chloroplasts and mitochondria in bundle sheath cells, and Rubisco and PEPC regulation (Sage et al. 2014). In grasses, no C_4_ plant is reported so far in the BEP (Bambusoideae, Ehrhartoideae, Pooideae) clade; the genus, *Oryza*, belongs to this clade (Grass Phylogeny Working Group II, 2012). However, many members of BEP clade are reported as having C_4_-like leaf structure, and *O. coarctata* is currently classified in this group (Christin et al. 2013). In addition, C_4_ plants usually have thin leaves so that the mesophyll and bundle sheath cells can coordinate closely to assure efficiently C_4_ photosynthesis (Ghannoum et al. 2005). *O. coarctata* has developed a minor vein on top of the major vein that shortens the distance between mesophyll cells on the adaxial side and vascular system, but this feature is not found in other *Oryza* species (Chatterjee et al. 2016). The *A/Ci* curve and CO_2_ compensation point indicate that the photosynthetic activities of *O. coarctata* are responsive to fluctuating CO_2_ concentration without having true C_4_ photosynthesis (Ku et al. 1983; Ueno et al. 2007; Schlüter et al. 2017; Monson and Jaeger 1991; Vogan and Sage 2012; Yorimitsu et al. 2019).

In C_4_ photosynthesis, NADP-ME, PEPC, and PPDK are responsible for oxaloacetate and phosphoenolpyruvate conversion in mesophyll cells and CO_2_ release in bundle sheath cells during the C_4_ cycle (Hibberd and Covshoff 2010). In C_4_ plants, Rubisco is responsible for the conversion of carbon into organic acid, and the two subunits of this enzyme, *RbcL*, and *RbcS,* are localized in chloroplasts and the nucleus, respectively (Berry et al. 2016). In this study, the expression of all C_4_ photosynthesis-related genes (*NADPME*, *PEPC*, *PPDK*, *RbcL*, and *RbcS*) was significantly higher in *O. coarctata* compared to IR64 and Pokkali. Although *O. coarctata* does not possess true C_4_ photosynthesis, higher gene expression, photosynthetic rates, and WUE as well as the unique morphology will be useful to understand the salinity tolerance mechanism in this halophytic wild rice.

## Methods and materials

### Greenhouse trial

Six wild rice species (*O. longiglumis* Jansen, *O. australiensis* Domin, *O. rufipogon* Griff*., O. latifolia* Desv., *O. officinalis* Wall. ex Watt and *O. coarctata* Roxb.), the *O. sativa* subspecies *indica* cultivar, IR64, and the landrace, Pokkali, were grown in a greenhouse at Western Sydney University, Hawkesbury Campus (33.62 °S, 150.75 °E). With the exception of *O. coarctata,* plants of the other species were raised from seed in sand for 14 days and then transferred to 9 L buckets filled with a loamy sandy soil. The remaining wild rice, *O. coarctata,* was prepared through vegetative propagation by separating newly emerged tillers with two leaves from parent plants. The young tillers were placed in Yoshida’s medium (Yoshida et al. 1971) for 14 days and transferred to the 9 L buckets containing the loamy sandy soil. The times of sowing and vegetative propagation of the wild and cultivated rice species were synchronized based on previous work to ensure that the plants were in the same growth stage during the measurements. The greenhouse was maintained with controlled day/night temperatures of 29 °C/24 °C and supplemental HPS lights with a 14/10 h day/night cycle. The relative humidity was maintained at 70–80%.

For each treatment and wild rice species/cultivated rice line, four replicates (4 buckets with 2 plants per bucket) were used for the different measurements. Water levels were maintained up to a height of 30 mm above the soil by daily watering with tap water. The initial EC of the soil was 0.25 dS m^− 1^. Incremental salinity treatments using NaCl commenced ~ 2 months after sowing with increments of 2 dS m^− 1^ per day until a final salinity of 10 dS m^− 1^ was reached. The final salinity treatment was maintained for six weeks before the collection of leaf samples for assessments. The EC values were monitored and maintained at the desired level throughout the growing season. After 42 days of salinity stress, plant height, tiller number and shoot biomass were measured. The 1st and 2nd fully expanded leaves were used for measurement of gas exchange, ion flux of mesophyll tissue, imaging of Na^+^ and ROS in mesophyll cells, chlorophyll content, nutrient analysis, and real-time PCR.

### Hydroponic trials

*O. coarctata* seedlings were prepared by vegetative propagation by separating freshly emerged tillers that had produced two leaves from their parent plants. The young tillers were placed in conical flasks containing a hydroponic solution consisting of Yoshida’s medium. Each flask was covered with aluminum foil to minimize exposure of the roots to sunlight. The solution was renewed every 3 days. After 2 weeks of growth, an incremental salinity stress treatment was applied through the subsequent addition to the medium of 25, 50, and 100 mM NaCl at 3-day intervals. After 1 month of salinity stress, Na^+^ localization in leaf tissue was measured using confocal imaging. The epidermal layer of the leaves was removed by using scalpel. For the 2 h salinity treatment, the exposed mesophyll tissue was submerged in the buffer solution with 50 or 100 mM NaCl.

### Gas exchange measurement

In the greenhouse trial, gas exchange was measured on the first, fully-expanded leaves using a *LI-6400XT* infrared gas analyser (LI-COR, Lincoln, USA) following Liu et al. (2017b); three leaves were randomly selected for measurement. In each measurement, the leaf was held in the chamber for 3–5 min before the reading was taken. The chamber conditions were as follows: flow rate, 500 mL min^− 1^, reference CO_2_ 400 μmol m^− 2^ s^− 1^; block temperature, 30 °C; light fluorescence, 1000 μmol m^− 2^ s^− 1^. The chamber reference humidity was maintained at ~ 60%.

For *A/Ci* curve measurements, the chamber conditions were set as follows: flow rate, 500 mL min^− 1^, block temperature, 30 °C; light fluorescence, 1000 μmol m^− 2^ s^− 1^, and reference CO_2_, 425 μmol m^− 2^ s^− 1^. Prior to the measurement, the selected leaf was held in the chamber for 20 min. Net CO_2_ assimilation rate was recorded at each of the following incremental ambient CO_2_ concentrations: 425, 600, 800, 1000, 1250, 1500, 1750 and 2000 μmol m^− 2^ s^− 1^. The readings at lower ambient CO_2_ concentrations of 350, 250, 150, 100 and 50 μmol m^− 2^ s^− 1^ were taken after 20 min adaptation at 425 μmol m^− 2^ s^− 1^ following the 2000 μmol m^− 2^ s^− 1^ treatment. The CO_2_ compensation point was measured from an *A/Ci* curve according to Laisk (1977).

### Chlorophyll content

Chlorophyll contents were determined as per Lichtenthaler and Buschmann (2001) with slight modifications. Ten milligrams of homogenised leaf samples were placed in Eppendorf tubes containing 1 mL 80% acetone and incubated in the dark overnight. Absorbance values were obtained at 470, 649, and 664 nm using a spectrophotometer.

### Leaf K^+^ and Na^+^ contents

K^+^ and Na^+^ contents in leaves were measured using a flame photometer (Jenway PFP7, John Morris, Australia) using a modified method based on Chen et al. (2007). The 1st and 2nd fully expanded leaves of each plant were harvested and oven-dried for 2 days. The dried leaf samples were finely ground and mixed using a Retsch Mixer Miller 400. Each sample (~ 50 mg) was digested in 4 mL of concentrated HNO_3_ (69%) in a boiling water bath until the sample solution was clear. The solutions were then diluted with 100 mL MilliQ water before measurement.

### Ion flux measurements

As leaf photochemistry is ultimately linked to ionic conditions in leaf mesophyll (Pottosin and Shabala 2016; Pan et al. 2020), steady-state net K^+^, Na^+^, Cl^−^ and Ca^2+^ fluxes were measured from leaf mesophyll cells of 1st fully expanded leaves using MIFE. Microelectrode preparation and mesophyll isolation protocols were as per Shabala et al. (2012). Prior to the measurement, leaf samples (20 × 20 mm^2^) were clamped in Perspex measuring chambers and submerged with standard MIFE solution (0.5 mM KCl, 0.1 mM CaCl_2_) and allowed to adapt to the new ionic environment for at least 1 h. Steady-state ion fluxes were then measured for ~ 5 min from each sample; there were 4–6 replicates for each combination of species and treatment. Net ion fluxes were calculated using MIFEFLUX software based on the ion concentration gradient recorded between two positions (see Shabala et al. (1997) for a theory and details).

### Confocal microscopy

The production of ROS and Na^+^ accumulation in mesophyll cells were measured using confocal microscopy according to Wang et al. (2016a). 5-(and 6-) chloromethyl-2′,7′-dichlorodihydrofluorescein diacetate, acetyl ester (CM-H_2_DCFDA) was employed to monitor cellular oxidative stress, and CoroNa Green AM was employed to localize Na^+^ distribution in leaf mesophyll tissues. The epidermal layer of the 1st fully expanded leaf was removed by using scalpel prior to incubation in a buffer solution (10 mM KCl, 5 mM Ca^2+^-MES, pH 6.1) with 20 μM of CM-H_2_DCFDA or 50 μM CoroNa Green for 1 h in the dark. ROS and Na^+^ were measured using an upright laser scanning confocal microscope (Leica SP5, Germany) with 50× objective lens [laser power: 10%, excitation laser: 488 nm (20%), emission range: 505–550, filter: TP488/543/633]. Chloroplasts were localized by detecting auto-fluorescence of chlorophyll at an emission range between 680 and 720. ROS and Na^+^ fluorescence was then quantified on a cell-by-cell basis.

### Quantitative real-time PCR

Transcripts of the following C_4_ pathway-related genes were quantified using total RNA extracted from the first fully expanded leaf samples collected at 42 days after sowing: *NADPME, *(*PEPC, PPDK, RBCL* and *RBCS* and salinity tolerance related genes *NHX1*, *VHA-C*, *VPPase*, *HKT1;4*, *SOS1*, *SOS2*, *SOS3*, *-ATPaseAHA1*, *HAK1*and* HAK5* (Liu et al. 2014; Wang et al. 2016b). Reverse transcription was performed as per the manufacturer’s instructions (Bioline, Australia). Quantitative real-time PCR (qPCR) was performed using a Quantinova SYBR Green Kit (QIAGEN, USA) in a Rotor-Gene 3000 quantitative PCR thermoscycler (QIAGEN, USA). Relative gene expression was calculated using the comparative threshold cycle (C_t_) 2^-∆∆Ct^ method (Livak et al. 2001); *glyceraldehyde 3-phosphate dehydrogenase* (*GAPDH*) and *elongation factor 1-alpha* (*EF1A*) were used as the internal reference genes. The experiments were conducted with three biological replicates and three technical replicates. The primer pairs are listed in Supplementary Table S1.

### Bioinformatic analysis

Chloroplast genome sequences from *Oryza* and other plant species were retrieved from public databases and were used for phylogenetic analysis of the genetic variation in cultivated and wild rice; the chloroplast genomes of five eudicot species were used as the outgroup. The sequences of PEPC, a C_4_ key enzyme in C_3_ and C_4_ species used in this study, were retrieved from the Michigan State University Rice Genome Annotation Project Database (http://rice.plantbiology.msu.edu/index.shtml), NCBI Protein and Nucleotide BLAST (https://blast.ncbi.nlm.nih.gov/Blast.cgi) and Ensembl Plants (http://plants.ensembl.org/index.html). These sequences were aligned using CLUSTAL W (http://www.clustal.org/clustal2/). The phylogenetic relationships among the sequences were inferred using maximum likelihood in MEGA X (Kumar et al. 2018). The percentages of trees from 1000 bootstrap replications in which the associated taxa clustered together are shown next to the branches.

### Data analysis

Independent sample t-tests, ANOVA, Duncan’s multiple range tests (DMRTs), Pearson’s correlation was performed using IBM SPSS Statistics, Version 24 (IBM Corp. Release 2020). *O. coarctata* outperformed the other *Oryza* species in this study with respect to salinity tolerance resulting outlying data; therefore, it was excluded from the correlation analyses. The relative values of the parameters from the control and salinity treatments were calculated to evaluate the salinity sensitivity of the species/cultivars. The ranking was based on the homogenous groups identified by DMRT of the parameters listed in Supplemental Table S2. The ranking of the homogenous groups for each parameter started from 1 as the most sensitive group and increased based on the numbers of homogenous groups identified by DMRT analyses. Relative values that occurred in multiple groups were averaged (e.g., if the species was included in groups 1 and 2 it was then ranked as 1.5). The average score of all parameters was used for the final ranking.

## Supplementary Information

**Additional file 1: Fig S1.**
Effect of salinity on biomass, plant height, tiller numbers and total chlorophyll of cultivated and wild rice species after 42 days of 100 mM NaCl. Mean ± SE (*n* = 4). Different lowercase letters indicate significant differences at *P* < 0.05. **Fig. S2.** Confocal imaging of Na^+^ fluorescence in mesophyll cells of *O. coarctata* in response to Control, 2 h of direct exposure to 50 mM NaCl, 2 h of direct exposure to 100 mM NaCl and 1 week of 100 mM NaCl treatment through root. The mesophyll tissue without epidermal layer was directly exposed to salinity solution. Yellow colour represents Na^+^ signal and red colour represents chlorophyll signal. Data are mean ± SE (*n* = 5). Different lowercase letters indicate significant differences at P < 0.05 for each Duncan Group

**Additional file 2: Table S1.**
The primers for analysis of gene expression related to salinity tolerance and C_4_ photosynthesis in cultivated and wild rice species. **Table S2.** Salinity tolerance scores (high values indicate tolerance, low values sensitivity), based on the ranking of homogeneous groups assigned by Duncan’s Multiple Range tests, of cultivated and wild rice species based on analyses of physiological parameters. **Table S3.** Correlation analysis among the physiological parameters and gene expression in the cultivated and wild rice species

## Data Availability

The authors declare that all data supporting the findings of this study are available within the article and its supplementary information files.

## Abbreviations

CM-H_2_DCFDA: 5-(and 6-) chloromethyl-2′,7′-dichlorodihydrofluorescein diacetate, acetyl ester
NADPME: NADP-dependent malic enzyme
PEPC: phosphoenolpyruvate carboxylase
PPDK: pyruvate phosphate dikinase
RBCL: ribulose-bisphosphate carboxylase
RBCS: ribulose-1,5-bisphosphate carboxylase/oxygenase small subunit
GAPDH: glyceraldehyde 3-phosphate dehydrogenase
EF1A: elongation factor 1-alpha
HAK: high affinity potassium transporter
SOS: salt overly sensitive
NHX: sodium/hydrogen exchanger
VHA-C: V-type H^+^ATPase subunit C
