# Supplementary material for: Proto Kranz-like leaf traits and cellular ionic regulation are associated with salinity tolerance in a halophytic wild rice
Source: Stress Biol. 2022 Jan 27;2(1):8. doi: 10.1007/s44154-021-00016-z (PMC10441962; doi:10.1007/s44154-021-00016-z)
Supplement: Supplementary file 1 — Additional file 1: Fig S1. Effect of salinity on biomass, plant height, tiller numbers and total chlorophyll of cultivated and wild rice species after 42 days of 100 mM NaCl. Mean ± SE (n = 4). Different lowercase letters indicate significant differences at P < 0.05. Fig. S2. Confocal imaging of Na+ fluorescence in mesophyll cells of O. coarctata in response to Control, 2 h of direct exposure to 50 mM NaCl, 2 h of direct exposure to 100 mM NaCl and 1 week of 100 mM NaCl treatment through root. The mesophyll tissue without epidermal layer was directly exposed to salinity solution. Yellow colour represents Na+ signal and red colour represents chlorophyll signal. Data are mean ± SE (n = 5). Different lowercase letters indicate significant differences at P < 0.05 for each Duncan Group [file 44154_2021_16_MOESM1_ESM.pptx]

## Slide 1
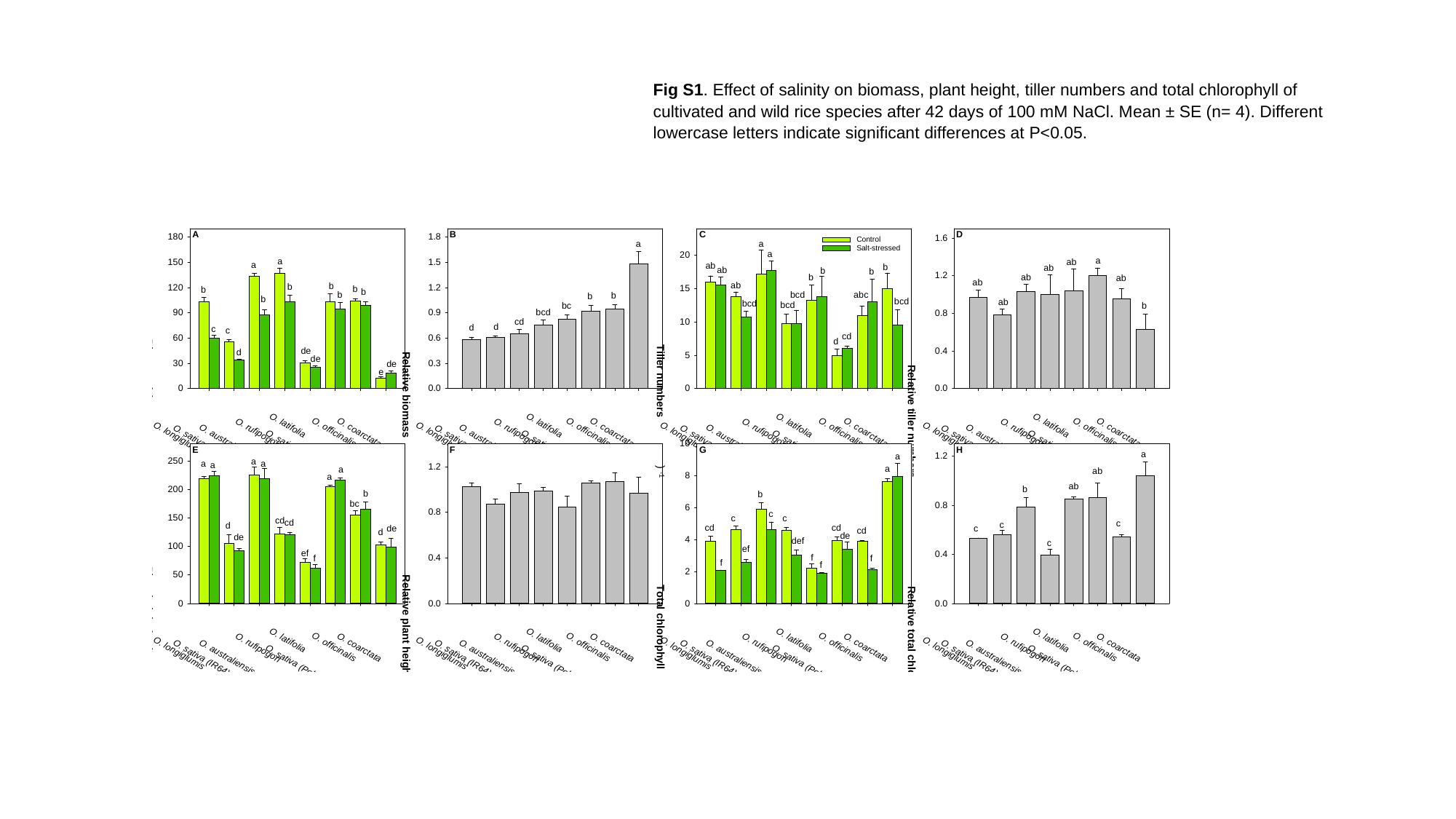

Fig S1. Effect of salinity on biomass, plant height, tiller numbers and total chlorophyll of cultivated and wild rice species after 42 days of 100 mM NaCl. Mean ± SE (n= 4). Different lowercase letters indicate significant differences at P<0.05.

## Slide 2
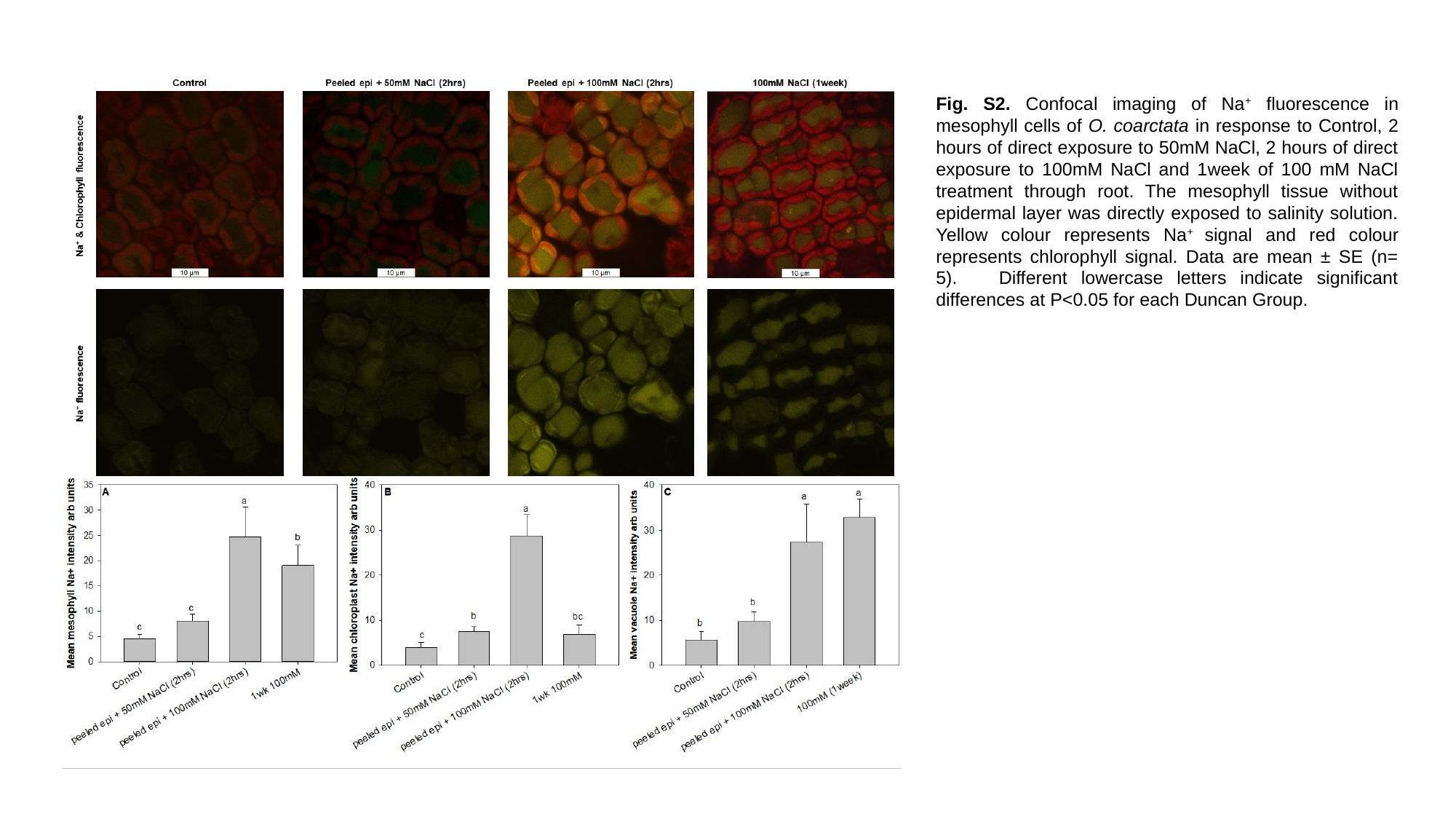

Fig. S2. Confocal imaging of Na+ fluorescence in mesophyll cells of O. coarctata in response to Control, 2 hours of direct exposure to 50mM NaCl, 2 hours of direct exposure to 100mM NaCl and 1week of 100 mM NaCl treatment through root. The mesophyll tissue without epidermal layer was directly exposed to salinity solution. Yellow colour represents Na+ signal and red colour represents chlorophyll signal. Data are mean ± SE (n= 5). Different lowercase letters indicate significant differences at P<0.05 for each Duncan Group.
