# Supplementary material for: Proto Kranz-like leaf traits and cellular ionic regulation are associated with salinity tolerance in a halophytic wild rice
Source: Stress Biol. 2022 Jan 27;2(1):8. doi: 10.1007/s44154-021-00016-z (PMC10441962; doi:10.1007/s44154-021-00016-z)
Supplement: Supplementary file 2 — Additional file 2: Table S1. The primers for analysis of gene expression related to salinity tolerance and C4 photosynthesis in cultivated and wild rice species. Table S2. Salinity tolerance scores (high values indicate tolerance, low values sensitivity), based on the ranking of homogeneous groups assigned by Duncan’s Multiple Range tests, of cultivated and wild rice species based on analyses of physiological parameters. Table S3. Correlation analysis among the physiological parameters and gene expression in the cultivated and wild rice species [file 44154_2021_16_MOESM2_ESM.docx]

**Table S1.** The primers for analysis of gene expression related to salinity tolerance and C­_4_ photosynthesis in cultivated and wild rice species.

| **Target Gene** | **Primer Name** | **Primer Sequence** |
| --- | --- | --- |
| ***HAK1*** | HAK1-F | CTCATCATCATCCCCATGCT |
|  | HAK1-R | CGGGAGATCAGCGAGTAGAG |
| ***HAK5*** | HAK5-F | TCGCATCTATCCAGAACACGTTGC |
|  | HAK5-R | TATGCATTGCCGATCTTGTCTGTAG |
| ***HKT1;4*** | HKT1;4-F | TTTGAGCTTTCTTCTCTCGGTGA |
|  | HKT1;4-R | TGAGCCTCCCAAAGAACATCAC |
| ***GORK*** | GORK-F | GATAGGGAGCTTGCAGTTGG |
|  | GORK-R | TCACAGCATGAATGTCACCA |
| ***SOS1*** | SOS1-F | AGATCGCGCTTACTCTTGCTGTC |
|  | SOS1-R | AGACCTCCAGTGCATCTTGTGC |
| **SOS2** | SOS2-F | ACTTAGCACTTTGGCCCAGAAAG |
|  | SOS2-R | ACCACATGACCAAACATCTGCTG |
| **SOS3** | SOS3-F | GAACATGTCACTTCCCTATTTGC |
|  | SOS3-R | GTCATGGGCTTCTGAATGCATT |
| ***NHX*** | NHX-F | CTGTCGTTCTTTTTAGCACTATGG |
|  | NHX-R | GGTGACAGGATGGCCTGA |
| ***AHA1*** | AHA1_ F | ACAGAACCTGGCTTGAGTGTG |
|  | AHA1_ R | GGGCAAGCAGCATAAACCCAAA |
| ***VHA-C*** | VHA-C-F | CTGTCGTTCTTTTTAGCACTATGG |
|  | VHA-C-R | GGTGACAGGATGGCCTGA |
| ***VPPase*** | VPPase_ F | ATGGCTCTCTTCGGAAGGGTTG |
|  | VPPase_ R | GTCACCGACATTGTCAGCAATCAC |
| ***Rubisco SS*** | Rubisco-SS-F | ACTCCAGCTTCGGCAACGTCAGCA |
|  | Rubisco-SS-R | ATACGGACGAATGCATCAGGGTAC |
| ***Rubisco LS*** | Rubisco-LS-F | ACACTGATATCTTGGCAGCATTCCGAG |
|  | Rubisco-LS-R | GTAGAGCGCGTAGGGCTTTGAAAC |
| ***PEPC*** | PEPC-F | GGAAGAAGATTTCTCCAGGAGAACCTTAC |
|  | PEPC-R | CAAGAACTGCTCGACATTGGTGTAAGTC |
| ***NADPME*** | NADPME-F | CTGATACAGTTTGAGGACTTCGCCAATC |
|  | NADPME-R | GAGCAATGAGTTCTGCAATACCAGTTCC |
| ***PPDK*** | PPDK-F | CACGAACGACCTTACGCAGA |
|  | PPDK-R | ACGGATCAAACGCCATCAC |
| ***G6PDH*** | G6PDH_F | AAGCCAGCATCCTATGATCAGATT |
|  | G6PDH_R | CGTAACCCAGAATACCCTTGAGTTT |
| ***ELF-a*** | ELF-a-F | CAGCAACTTGACTATGGATTGGTGGA |
|  | ELF-a-R | CATCCAGCACAAACATCTTAATGTGGTC |

**Table S2.** Salinity tolerance scores of cultivated and wild rice species based on the key physiological parameters.

| Salinity ranking | Biomass | Chl a | Chl b | ROS intensity | Na intensity | Na:K ratio | *A* | *E* | Average score |
| --- | --- | --- | --- | --- | --- | --- | --- | --- | --- |
| *O. coarctata* | 4 | 4 | 4 | 2 | 2 | 4 | 1.5 | 1 | 2.8 |
| *O. officinalis* | 3 | 1 | 2.5 | 2 | 3 | 4 | 2 | 2 | 2.4 |
| *O. sativa* L. Pokalli | 3 | 3.5 | 3 | 2 | 2.5 | 4 | 1.5 | 1 | 2.6 |
| *O. latifolia* | 2.5 | 3 | 3 | 2 | 2.5 | 4 | 1.5 | 1 | 2.4 |
| *O. australiensis* | 1.5 | 3 | 2.5 | 1.5 | 2 | 3 | 1.5 | 1 | 2.0 |
| *O. ruffipogon* | 2 | 2 | 2.5 | 2 | 2 | 3 | 1.5 | 1 | 2.0 |
| *O. sativa* L. IR64 | 1 | 1 | 1.5 | 1.5 | 1.5 | 1 | 1 | 1 | 1.2 |
| *O. longiglumis* | 1 | 1.5 | 1 | 1 | 1 | 2 | 1 | 1 | 1.2 |

|  | | Biomass | Height | Tiller numbers | ∆K^+^ flux | ∆Na^+^ flux | ∆Cl^-^ flux | ∆Ca^2+^ flux | mesophyll ROS | mesophyll Na+ | Na content | K content | Na:K ratio | *A* | *g_s_* | | *E* | chl a | chl b |  |  |  |  |  |  |  |  |  |  |  |  |  |  |  |  |  |  |  |  |  |
| --- | --- | --- | --- | --- | --- | --- | --- | --- | --- | --- | --- | --- | --- | --- | --- | --- | --- | --- | --- | --- | --- | --- | --- | --- | --- | --- | --- | --- | --- | --- | --- | --- | --- | --- | --- | --- | --- | --- | --- | --- |
| Biomass | | 1 |  |  |  |  |  |  |  |  |  |  |  |  |  | |  |  |  |  |  |  |  |  |  |  |  |  |  |  |  |  |  |  |  |  |  |  |  |  |
| Height | | 0.38 | 1 |  |  |  |  |  |  |  |  |  |  |  |  | |  |  |  |  |  |  |  |  |  |  |  |  |  |  |  |  |  |  |  |  |  |  |  |  |
| Tiller numbers | | 0.84* | 0.67 | 1 |  |  |  |  |  |  |  |  |  |  |  | |  |  |  |  |  |  |  |  |  |  |  |  |  |  |  |  |  |  |  |  |  |  |  |  |
| ∆K^+^ flux | | 0.89** | 0.24 | 0.80* | 1 |  |  |  |  |  |  |  |  |  |  | |  |  |  |  |  |  |  |  |  |  |  |  |  |  |  |  |  |  |  |  |  |  |  |  |
| ∆Na^+^ flux | | 0.32 | -0.36 | -0.09 | 0.24 | 1 |  |  |  |  |  |  |  |  |  | |  |  |  |  |  |  |  |  |  |  |  |  |  |  |  |  |  |  |  |  |  |  |  |  |
| ∆Cl^-^ flux | | 0.77* | 0.12 | 0.61 | 0.69 | 0.60 | 1 |  |  |  |  |  |  |  |  | |  |  |  |  |  |  |  |  |  |  |  |  |  |  |  |  |  |  |  |  |  |  |  |  |
| ∆Ca^2+^ flux | | -0.27 | -0.06 | 0.04 | -0.09 | -0.86* | -0.46 | 1 |  |  |  |  |  |  |  | |  |  |  |  |  |  |  |  |  |  |  |  |  |  |  |  |  |  |  |  |  |  |  |  |
| Mesophyll ROS | | 0.81* | 0.40 | 0.76* | 0.72 | 0.24 | 0.89** | -0.23 | 1 |  |  |  |  |  |  | |  |  |  |  |  |  |  |  |  |  |  |  |  |  |  |  |  |  |  |  |  |  |  |  |
| Mesophyll Na+ | | -0.90** | -0.25 | -0.79* | -0.81* | -0.51 | -0.87* | 0.38 | -0.75 | 1 |  |  |  |  |  | |  |  |  |  |  |  |  |  |  |  |  |  |  |  |  |  |  |  |  |  |  |  |  |  |
| Na content | | -0.86* | -0.36 | -0.89** | -0.97** | -0.12 | -0.73 | 0.00 | -0.80* | 0.81* | 1 |  |  |  |  | |  |  |  |  |  |  |  |  |  |  |  |  |  |  |  |  |  |  |  |  |  |  |  |  |
| K content | | 0.90** | 0.51 | 0.90** | 0.73 | 0.07 | 0.59 | -0.05 | 0.65 | -0.85* | -0.75 | 1 |  |  |  | |  |  |  |  |  |  |  |  |  |  |  |  |  |  |  |  |  |  |  |  |  |  |  |  |
| Na:K ratio | | -0.86* | -0.37 | -0.91** | -0.86* | -0.21 | -0.86* | 0.09 | -0.86* | 0.91** | 0.94** | -0.82* | 1 |  |  | |  |  |  |  |  |  |  |  |  |  |  |  |  |  |  |  |  |  |  |  |  |  |  |  |
| *A* | | 0.56 | 0.25 | 0.50 | 0.69 | 0.58 | 0.67 | -0.60 | 0.53 | -0.67 | -0.68 | 0.33 | -0.62 | 1 |  | |  |  |  |  |  |  |  |  |  |  |  |  |  |  |  |  |  |  |  |  |  |  |  |  |
| *g_s_* | | 0.49 | 0.19 | 0.55 | 0.73 | 0.29 | 0.37 | -0.21 | 0.24 | -0.60 | -0.69 | 0.42 | -0.57 | 0.83* | 1 | |  |  |  |  |  |  |  |  |  |  |  |  |  |  |  |  |  |  |  |  |  |  |  |  |
| *E* | | 0.70 | 0.66 | 0.74 | 0.40 | -0.22 | 0.39 | 0.08 | 0.63 | -0.52 | -0.47 | 0.83* | -0.57 | -0.05 | -0.09 | | 1 |  |  |  |  |  |  |  |  |  |  |  |  |  |  |  |  |  |  |  |  |  |  |  |
| Chl a | | 0.22 | -0.15 | 0.39 | 0.54 | 0.02 | 0.42 | -0.45 | 0.24 | -0.37 | -0.64 | 0.16 | -0.55 | 0.54 | 0.74 | | -0.26 | 1 |  |  |  |  |  |  |  |  |  |  |  |  |  |  |  |  |  |  |  |  |  |  |
| Chl b | | 0.55 | -0.29 | 0.41 | 0.87* | 0.61 | 0.79* | -0.87* | 0.51 | -0.73 | -0.71 | 0.36 | -0.71 | 0.72 | 0.69 | | -0.10 | 0.78* | 1 |  |  |  |  |  |  |  |  |  |  |  |  |  |  |  |  |  |  |  |  |  |
|  |  | | | | | | | | | | | | | | |  | | | | |  |  |  |  |  |  |  |  |  |  |  |  |  |  |  |  |  |  |  |  |
|  |  | | | | | | | | | | | | | | |  | | | | |  |  |  |  |  |  |  |  |  |  |  |  |  |  |  |  |  |  |  |  |
|  |  | | | | | | | | | | | | | | |  | | | | |  |  |  |  |  |  |  |  |  |  |  |  |  |  |  |  |  |  |  |  |
|  |  | | | | | | | | | | | | | | |  | | | | |  |  |  |  |  |  |  |  |  |  |  |  |  |  |  |  |  |  |  |  |
|  |  | | | | | | | | | | | | | | |  | | | | |  |  |  |  |  |  |  |  |  |  |  |  |  |  |  |  |  |  |  |  |

**Table S3:** Correlation analysis among the physiological parameters and gene expression in cultivated and wild rice species.
